# The Prevalence of Depression among Family Caregivers of Children with Intellectual Disability in a Rural Setting in Kenya

**DOI:** 10.1155/2011/534513

**Published:** 2011-10-02

**Authors:** Margaret Njeri Mbugua, Mary W. Kuria, David M. Ndetei

**Affiliations:** ^1^Department of Psychiatry, University of Nairobi, P.O. BOX 365-00621, Village Market, Nairobi, Kenya; ^2^Department of Psychiatry, University of Nairobi and Africa Mental Health Foundation, P.O. BOX 48423-00100, Nairobi, Kenya

## Abstract

Caregivers of children with intellectual disability have a great responsibility that may be stressful. The psychological well-being of the care giver may affect the quality of care given to children with intellectual disability. *Objective*. The objective of the study was to determine the risk of depression in caregivers of children with intellectual disability. *Setting*. The study was conducted at Gachie Catholic Parish, Archdiocese of Nairobi (Kenya). *Design.* Cross sectional, descriptive study. *Method*. The study was conducted among 114 caregivers registered at the Gachie Parish program (in Kenya) for the intellectual disabled children. A researcher-designed social demographic questionnaire and the Beck depression inventory were administered to those that met the inclusion criteria. *Results*. Seventy-nine percent (79%) of the caregivers were at risk of clinical depression. *Conclusion*. Majority of the caregivers of children with intellectual disability were at risk of developing clinical depression.

## 1. Introduction

There is an increased demand on the care givers of children with developmental disability [1] especially when it is intellectual in nature. Intellectual disability is associated with poor communication, academic and social skills that make the child more dependent on the caregiver than the normal child. 

In the past (in Kenya), the extended family would be available to provide care to an intellectually disabled child easing the burden of care expected from the nuclear family. However, in recent times, there is a shift from extended family to nuclear family. In addition, over the past 2 decades, family units have become smaller [2] and the rate of marriage break down has increased [3]. Though the magnitude of the responsibility depends on the level of intellectual disability, it is greater for those in small nuclear families. 

It is also important to note that the economic situation in Kenya dictates that people work long hours with little pay especially if they are in nonskilled employment. This coupled with the fact that there are few institutions or organizations that school or take care of the intellectually disabled makes the role of caregivers enormous. The caregivers are usually the mother of the child, elderly family members, or the unemployed members of the family. Such people do not normally plan to be caregivers [4] but find the need unavoidable. In addition, the caregivers do not receive preparation for this role, and, in the process of engaging in the same, they later on find it very demanding [4]. Unlike other carers, the caregiver may be deprived of privileges, rights, and respect that go with other careers [3]. Further, there is lack of career progression, and the individual may continue to work involuntarily [3]. In the majority of cases in Kenya, the caregiver receives no financial assistance due to the poor socioeconomic status of the people. The caregiver may experience lack of control of what happens in their lives. A sense of mastery is associated with good physical and psychological health of an individual [5–7]. This may be absent in some of the caregivers. The relationship between caregiving and health is described generally in terms of stress. Stressors in the context of caregiving are the difficult circumstances and problems. Such demands and obstacles exceed or push to the limit the caregivers' capacity to adapt [2].

5 factors that have been shown to contribute to poor health outcomes of the caregivers include child's behavior [8], child's temperament [9], severity of the disability [10–14], low self-esteem [15, 16], and poor social support [8, 10, 17, 18]. Literature indicates that the level of communication ability, specific cognitive or sensory impairments, sex, and age of the patient may be relevant to the mental health of the caregiver [11, 19–22].

 Factors that lead to low psychological distress in mothers of intellectual disabled persons include, a positive belief system and “noncritical family network” [11]. Other coping factors include stress management, family functioning [23–26], and support from the spouse [27–29]. Caregivers health can be improved by specific stress management strategies [11, 30].

## 2. Materials and Method

The cross-sectional descriptive study was conducted in Gachie Parish in Kiambu (Kenya) between September and November 2007. Gachie Parish is located 15 Km north of Nairobi and consists of eight churches with a population of 10,000 followers. The location has both local residents who do peasant farming as well as tenants who work in Nairobi city. It is a cosmopolitan location with people of both low and high socioeconomic status. Parish has a database of all the children in Parish who have intellectual disabilities. This data was collected in early 2007 by the Parish leaders in an attempt to offer assistance to the needy cases. All the caregivers of 150 registered intellectually disabled persons were requested to participate in the study. One hundred and fourteen fulfilled the inclusion criteria which included caregiver age between 18 and 70 years, informed consent, and diagnosis of intellectual disability having been done before the child attained the age of 18 years. Thirty-six persons in the database did not participate in the study because they did not fulfill the inclusion criteria. 

The participants were assured of confidentially. Participation was voluntary, and other ethical considerations were fulfilled. Ethical approval was obtained from Kenyatta National Hospital, University of Nairobi Ethical and Research committee, and permission to perform the study was obtained from the Department of Psychiatry, University of Nairobi. 

The enrolled participants were subjected to a sociodemographic questionnaire to collect data on the age, sex, marital status, religion, education status, and occupation. The Beck Depression inventory was administered to collect data on the risk of depression. The Kiswahili version (provided by the 3rd author) of the Beck Inventory was administered by the 1st author who was trained by the second author. Kiswahili is the national language in Kenya and is well understood by the majority of Kenyans. This is an inventory with twenty-one multiple-choice self-report inventory questionnaire and is one of the most widely used instruments for measuring risk of depression. The questionnaire is designed for adults aged 17–80 years. 

The Father in charge received an advanced written explanation highlighting what the study was all about. He thereafter made an announcement in the church, and the caregivers gathered at the parish hall at the given date. After introduction, the principal investigator explained the details about the study, the benefits and the risks, and the fact that it was a voluntary exercise. Those that consented after the explanation signed a consent form, while those unwilling to participate were allowed to leave. The consenting participants completed sociodemographic questionnaires and a Kiswahili version of the Beck Inventory. Those that needed assistant to complete the questionnaires were helped by the principal investigator and research assistants. No data related to the severity of the intellectual disability of the persons who were under the care of the caregiver was collected since this was not within the scope of the current study.

## 3. Data Analysis

The data collected was entered in an Excel spreadsheet, cleaned, and analyzed using the statistics package for social sciences (SPSS) version 12. 

Multivariate regression analysis was done, and the results were presented in tables.

## 4. Results

All the caregivers were Kenyans of African origin, and the children under their care were aged between one and eighteen years of age. 

Majority of caregivers were aged between 21 years and 45 years of age. All the caregivers were Christians with 89.5% being female. 

Majority of caregivers were married, unemployed, and had primary school education (this is the first eight years of school while secondary education is the 9th–12th year of education). Results are shown in Table 1. 

The majority of caregivers (79%) were at risk of having clinical depression as per result table showing percentages of likely severe, moderate, and mild depression. Results are shown in Table 2. 

Sociodemographic factors that were associated with the risk of depression included gender, unemployment, primary education, married status, and the age of caregiver. The results are shown in Table 3. 

## 5. Discussion

In the current study, 79% of the caregivers were at risk of clinical depression as derived from Beck Inventory score. This finding is consistent with that of Turner and Sanders [31]. All the caregivers had not previously been diagnosed by a clinician for depression. Those found with the risk of clinical depression were referred to Kenyatta National Hospital for further clinical evaluation and treatment. Various factors may contribute to vulnerability of the caregiver to depression, and of importance is financial lack as the caregiver is unable to engage in income generating activity. In addition, social isolation, loss of previously close friendships, and stigma associated with taking care of intellectually disabled person may further predispose the caregiver to the risk of depression. 

The sociodemographic characteristic of the caregiver shows that there was a statistically significant association between unemployment and risk of depression. Unemployed caregivers have little or no income. Further, the burden related to financial costs is aggravated by insufficient public resources in place at the community level, such as lack of schools for the intellectually disabled and proper health facilities to meet their health needs. In Kenya, special schools or classes and special teachers for the intellectually disabled persons are few and apart with some regions having none. 

The married caregivers had statistically significant higher levels of risk of depression than the singles, divorced, or separated because in the African culture, intellectual disability is associated with stigma and, hence, the risk of higher levels of depression among the married. Nearly half (44.7%) of the depressed caregivers were married. This may be due to lack of emotional support from the spouse. The divorced/separated and single may on the other hand have adapted coping strategies to deal with the situation. Frey et al. [32] had similar findings in their study. 

The finding that gender was significantly associated with risk of depression is in keeping with findings of other studies [33]. The women are responsible for the emotional care of the children. In the African context, it is more acceptable for the woman to take up the role of the caregiver. This may result in low self-esteem and loss of self that may be associated with maternal depression due to subjective caregiving burden among them [33]. 

All the participants were Christians which reflects religious background of the study area.

## 6. Limitations of the Study

1. Preexisting depression was not assessed and may have had an influence on the findings.
2. This study was researched in a single locality and, hence, it would be difficult to ascertain if the risk of depression of the caregivers would be the same or different from other localities vis-à-vis urban and rural settings.

## 7. Conclusion

Beck Inventory scores indicated that majority of caregivers of children with intellectual disability had scores indicating risk of mild, moderate, and severe depression. The current study shows that caregivers of persons with intellectual disability are likely to be at risk of depression. There is therefore need to avail a support system to prevent or reduce the risk of depression in the caregivers of intellectually disabled children.

## Figures and Tables

**Table 1:** Social demographic characteristics of caregivers.

		Number	Percentage
Age group	21–30	34	23.1%
	31–45	40	31.5%
	41–65	30	26.3%
	65–70	12	10.5%

	Total	114	100%

Gender	Male	12	10.5%
	Female	102	89.5%

	Total	114	100%

Religion	Catholic	61	53.5%
	Protestant	53	46.5%

	Total	114	100%

Education	Informal	18	15.8%
	Primary	66	57.9%
	Secondary	30	26.3%

	Total	114	100%

Marital status	Single	31	27.2%
	Married	63	55.3%
	Divorced	7	6.1%
	Separated	13	11.4%

	Total	114	100%

Occupation	Not employed	84	73.7%
	Business	18	15.8%
	Student	12	10.5%

	Total	114	100%

**Table 2:** Risks of depression levels in the caregivers.

N114	Frequency	Percentage
Minimum depression	24	21.1
Mild depression	30	26
Moderate depression	24	21
Severe depression	36	31.6

Total	114	100

**Table 3:** Social demographic correlates of depression.

Age of caregiver and depression	25–30	24.5	28	0.001
	31–45	26.3	30	“
	46–65	23.7	27	“
	>66	4.4	5	“

Religion and depression	Protestants	46.6	53	0.003
	Catholics	32.4	37	“

Gender and depression	Male	10.6	12	0.02
	Female	68.5	78	

Marital status and depression	Single	21.9	25	“
	Married	44.7	51	“
	Divorced	6.2	7	“
	Separated	6.2	7	“

Level of education and depression	Informal	10.6	12	0.000
	Primary	52.6	60	“
	Secondary	15.8	18	“

Employment status and depression	Unemployed	63.1	72	0.000
	Business	5.3	6	“
	Student	10.5	12	“

Other sibling in the family and depression		78.9	90	0.001

“ means the significant is the same as the first one in each particular column.
